# The Response of Extracellular Polymeric Substances Production by Phototrophic Biofilms to a Sequential Disturbance Strongly Depends on Environmental Conditions

**DOI:** 10.3389/fmicb.2021.742027

**Published:** 2021-10-11

**Authors:** Emilie Loustau, Joséphine Leflaive, Claire Boscus, Quentin Amalric, Jessica Ferriol, Olga Oleinikova, Oleg S. Pokrovsky, Elisabeth Girbal-Neuhauser, Jean-Luc Rols

**Affiliations:** ^1^Laboratoire Ecologie Fonctionnelle et Environnement, Université de Toulouse, CNRS, Toulouse INP, Université Toulouse 3 – Paul Sabatier (UPS), Toulouse, France; ^2^LBAE, Université de Toulouse, Université Toulouse 3 – Paul Sabatier (UPS), Auch, France; ^3^GET, Université de Toulouse, CNRS, IRD, Université Toulouse 3 – Paul Sabatier (UPS), Toulouse, France; ^4^BIO-GEO-CLIM Laboratory, Tomsk State University, Tomsk, Russia

**Keywords:** EPS production, phototrophic biofilm, resistance, recovery, photosynthetic efficiency

## Abstract

Phototrophic biofilms are exposed to multiple stressors that can affect them both directly and indirectly. By modifying either the composition of the community or the physiology of the microorganisms, press stressors may indirectly impact the ability of the biofilms to cope with disturbances. Extracellular polymeric substances (EPS) produced by the biofilm are known to play an important role in its resilience to various stresses. The aim of this study was to decipher to what extent slight modifications of environmental conditions could alter the resilience of phototrophic biofilm EPS to a realistic sequential disturbance (4-day copper exposure followed by a 14-day dry period). By using very simplified biofilms with a single algal strain, we focused solely on physiological effects. The biofilms, composed by the non-axenic strains of a green alga (*Uronema confervicolum*) or a diatom (*Nitzschia palea*) were grown in artificial channels in six different conditions of light intensity, temperature and phosphorous concentration. EPS quantity (total organic carbon) and quality (ratio protein/polysaccharide, PN/PS) were measured before and at the end of the disturbance, and after a 14-day rewetting period. The diatom biofilm accumulated more biomass at the highest temperature, with lower EPS content and lower PN/PS ratio while green alga biofilm accumulated more biomass at the highest light condition with lower EPS content and lower PN/PS ratio. Temperature, light intensity, and P concentration significantly modified the resistance and/or recovery of EPS quality and quantity, differently for the two biofilms. An increase in light intensity, which had effect neither on the diatom biofilm growth nor on EPS production before disturbance, increased the resistance of EPS quantity and the resilience of EPS quality. These results emphasize the importance of considering the modulation of community resilience ability by environmental conditions, which remains scarce in the literature.

## Introduction

Understanding and predicting how communities respond and resist to disturbances in terms of composition or function are current concerns for microbial ecologists (Gonzalez et al., 2012). Ecological disturbance is defined as a causal event inducing a perturbation in a community (Rykiel, 1985). Many definitions have been proffered for the resilience of communities exposed to a disturbance. We consider here, following the recommendation by Hodgson et al. (2015), the resilience as the ability of a system to persist or maintain a function in the face of disturbance. The two measurable components of resilience are the resistance, that is the degree to which a community remains unchanged when it is subjected to a disturbance, and the recovery, that is the ability of a system to return to pre-disturbance levels (Hodgson et al., 2015; Oliver et al., 2015). Consequently, a resilient system is able to minimize the impact of the disturbance and has the ability to resume functioning under changing conditions. The resilience of microbial communities is crucial to preserve the ecological services they provide. With global change, the intensity and frequency of disturbances are likely to increase (IPCC, 2013), threatening the upholding of ecological services. By impacting the ability of microbial communities to resist to and recover after disturbances, small modifications of the environmental conditions may also represent an underestimated threat. The aim of this study was to assess in what extent the environmental conditions could influence the response of a microbial community to a complex disturbance.

Very simplified phototrophic biofilms were chosen as model of microbial community. In natural environment, biofilms are complex microbial communities composed of heterotrophic and phototrophic, eukaryotic and prokaryotic microorganisms embedded in a protective matrix of extracellular polymeric substances (EPS) (Lock, 1993). The functions fulfilled by phototrophic biofilms, such as photosynthesis, primary production, oxygen production, nutrients uptake, and contaminants removal are at the base of key ecological functions of fluvial ecosystems (Lawrence et al., 2001; Battin et al., 2003, 2016; Corcoll et al., 2012). Phototrophic biofilms are often exposed to chemical disturbances when freshwater ecosystems receive organic or inorganic pollutants (pesticides, organic solvents, heavy metals) through agriculture, domestic, and industrial uses (Segner et al., 2014). In addition to these multiple chemical stresses, the biofilms are also exposed to desiccation when the flow decreases. This stress is particularly severe in intermittent rivers which completely dry out part of the year (Datry et al., 2017). It is generally agreed that climate change will lead to more intense summer drought, higher temperatures, and more frequent episodes of intense rainfall (Acuna, 2010; Sabater et al., 2016). Hydrological and chemical stresses may affect aquatic systems either simultaneously or successively. For example, a decrease of flow before full desiccation can induce an increase in the concentration of riverine solutes (Gomez et al., 2017).

Light intensity, temperature, and nutrients are among the main environmental factors that affect the growth of phototrophic microbial communities (Lear et al., 2008; Sabater et al., 2016; Bengtsson et al., 2018). Light is the principal energy source fueling biofilm primary production: a modification of light intensity affects biofilm growth, its physiology and the community composition (Sabater et al., 2002). Temperature increase can change algal diversity (Stevenson et al., 1996). Furthermore, temperature has a marked influence on chemical toxicity (Holmstrup et al., 2010). Lambert et al. (2016) and Pesce et al. (2018) showed that temperature can modulate phototrophic biofilm response to chronic Cu exposure: an increase in temperature reduced Cu effects on algal biomass, photosynthetic efficiency, and diatom composition. The availability and the concentration of nutrients also play an important role on changes in the structure of phototrophic biofilm communities with consequences on the sensitivity of biofilms to disturbances. For instance, as inorganic nutrient concentrations increase, the proportion of species tolerant to organic pollution increases whereas the abundance of sensitive species declines (Delgado et al., 2012). This is particularly true for phosphorus (P) which is a limiting nutrient in many aquatic ecosystems (Schindler, 1977; Schindler et al., 2008). Increasing P concentration led to a significant shift in the biofilm community composition; these changes being more pronounced in diatom- than in cyanobacteria-dominated biofilm (Leflaive et al., 2015).

One essential component of biofilms is the EPS matrix which provides a cohesion to the assemblage of organisms. EPS are produced by bacteria and microalgae through excretion, sorption, or cell lysis, and are mainly composed by polysaccharides and proteins with a certain amount of lipids, DNA and humic substances (Loustau et al., 2018). EPS create strong gradients within the biofilm by limiting access to nutrients, oxygen and light for the cells located far from the biofilm surface (Hill, 1996). Besides other functions (adhesion of microorganisms on immersed substratum, resource for the growth of heterotrophic microorganisms), the EPS matrix protects the cells from desiccation and other environmental stresses (pollutants, UV radiation, etc.), and adsorbs and accumulates organic pollutants or metal cations present in the water column (Flemming et al., 2016). EPS are certainly involved in the ability of phototrophic microorganisms to cope with disturbance and tolerate stresses, as it has been suggested for heavy metals, nanoparticles (Kroll et al., 2014), salinity (Shetty et al., 2019), or drought (Sasaki et al., 2020). Since EPS production is quantitatively and qualitatively affected by environmental conditions such as temperature and light intensity (Wolfstein and Stal, 2002; Di Pippo et al., 2012) or the presence of nanoparticles (Verneuil et al., 2015), our main hypothesis is that slight variations in environmental conditions in terms of light, temperature or nutrient concentration may impact the resilience of EPS quality and quantity to a disturbance. To test this hypothesis, we monitored the production and composition of EPS in response to a complex disturbance, in different environmental conditions of light, temperature, and nutrients. Biofilms composed by one phototrophic microorganism and the associated bacterial communities were produced in artificial streams. Such simplified biofilms were chosen in order to mainly focus on direct effects on algal physiology rather than on indirect effects through modifications of algal composition. The sequence of disturbance was chosen to be representative of flow decrease and cessation in a river: an increase in pollutant concentration followed by desiccation. Copper, commonly found is small intermittent rivers in vineyard areas (Montuelle et al., 2010), was chosen here as a model of pollutant. Our hypotheses are that (i) the environmental conditions influence the resilience of the quality and quantity of EPS when the biofilms are exposed to a disturbance and (ii) the more an environmental parameter affects the biofilm growth, the more it affects the resilience of EPS.

## Materials and Methods

### Pre-culture of Microorganisms

The diatom *Nitzschia palea* (Kützing) W. Smith and the green alga *Uronema confervicolum* Lagerheim, isolated from phototrophic biofilms in the rivers Tarn and Garonne (Southwestern France), were used as the main phototrophic organisms for the two different biofilms. They were maintained in non-axenic conditions in the algae collection of the laboratory “Ecologie Fonctionnelle et Environnement” at 18°C under white light (30 ± 5 μmol photons s^–1^m^–2^) with light/dark periods of 16/8 h in Combo medium (Kilham et al., 1998). Individual pre-cultures were prepared for 21 days in 500-mL Erlenmeyer flasks and the biomass was homogenized using an Ultra-Turrax disperser (T25, Janke-Kunkel, 13,500 rpm, 1 min) before inoculation in the hydraulic mini-channels.

### Experimental Setup

**FIGURE 1:**
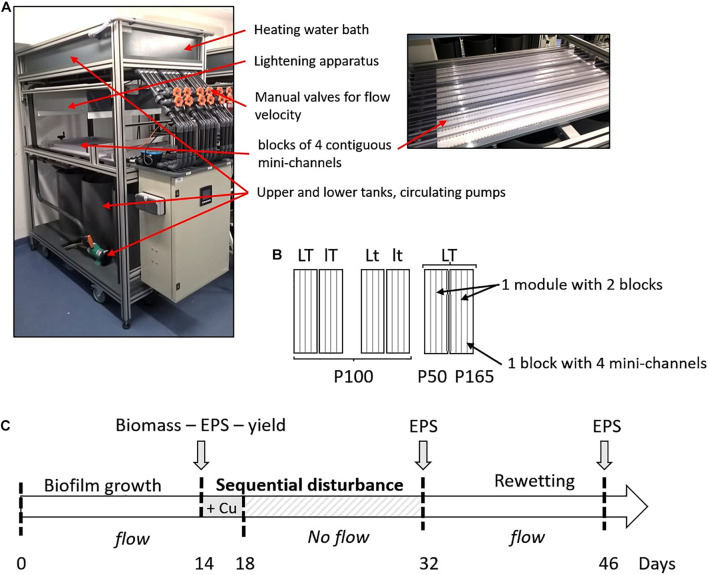
**(A)** Photographs of one module with a detail view of the mini-channels. **(B)** Experimental design applied for the two biofilms. The experimental conditions were the same in the four mini-channels of one block. Six different conditions were tested with two levels of temperature (*T* = 22°C or *t* = 19°C), light intensity (*L* = 70 μmol s^–1^m^–2^ or *l* = 30 μmol s^–1^m^–2^) and P concentration (P100 corresponds to the initial P concentration of 5.23 mg L^–1^ in the culture medium, P50 with a decrease of 50% and P165 with an increase of 165%). **(C)** Experimental setup with the step of inoculation of the mini-channels at day 0, the addition in the culture medium of Cu for a final concentration of 50.3 μg L^–1^ at day 14, followed by a 14-day dry period, and finally a 14-day rewetting.

Very simplified biofilms (one algal strain and the associated bacteria) were grown in several hydraulic mini-channels described in Loustau et al. (2019). Three modules were used, each one composed of two blocks corresponding to two separated closed circulating loops of Combo medium for the supply of four mini-channels in each block (Figure 1A). Only three mini-channels in each block were sampled for this study. Two successive experiments were performed, one for each type of biofilm cultivated on eighty supports (polyoxymethylene coupons, size 29 mm × 10 mm × 5 mm) transversally placed along each mini-channel. A flow velocity of 0.4 m s^–1^ allowing to create a homogeneous turbulent flow regime on the surface of coupons, and a circadian cycle of light/dark periods of 16/8 h were applied. A pH of 8 ± 0.1 (pH meter Multi 3430 SET C) was observed during the light period. Biofilms were cultivated with a combination (Figure 1B) of two light intensities (70 μmol photons s^–1^m^–2^, *L* - high light intensity or 30 μmol photons s^–1^m^–2^, *l* - low light intensity) measured with a flat quantum sensor (model LI-189, LI-COR, Inc., Lincoln, Nebraska) and two temperatures of the circulating water (22°C, *T* - high temperature or 19°C, *t* - low temperature). Two additional conditions at high temperature and high light intensity were obtained by modifying initial P concentration compared to the initial P concentration of the culture medium (5.23 mg L^–1^, *P100* - 100%): a higher concentration of 8.71 mg L^–1^ (*P165* – increase of 165%) and a lower concentration of 2.61 mg L^–1^ (*P50* - decrease of 50%). For each condition, a block was inoculated (Figure 1C). The six culture conditions were then: LTP100, LtP100, lTP100, ltP100, LTP50, and LTP165.

After 14 days of biofilm growth, the physiological state of biofilm was assessed with maximum quantum yield measurements directly inside the mini-channels, and some coupons were randomly sampled for the determination of biomass and EPS (amount and composition).

After the sampling at day 14, copper (CuSO_4_) was added in the circulating nutrient medium to reach an increase of the initial Cu concentration by 50 μg L^–1^. Due to Cu presence in Combo culture medium (0.3 μg L^–1^), the initial Cu concentration corresponded to 50.3 μg L^–1^. Dissolved Cu^2+^(aq) speciation in the presence of cell exometabolites and organic ligands of the nutrient medium was assessed by using the Visual MINTEQ computer code (Gustafsson, 2011, version 3.1 for Windows, October 2014) in conjunction with a database and the NICA-Donnan humic ion binding model. Thermodynamic calculation demonstrated that Cu^2+^(aq) speciation was dominated by EDTA complexes in the form of Cu-anions, with negligible role of other organic complexes from nutrient media constituents.

At day 18, the mini-channels were quickly dried-out by removing the water in each block. The biofilms were then kept at room temperature (17°C). After 14 days of dry period (day 32), the mini-channels were re-filled with fresh culture medium with corresponding initial P concentration (*P50*, *P100*, or *P165*) for another 14-day rewetting period (until day 46).

Before the disturbance event (day 14), ten coupons were randomly selected and collected per mini-channel for the biomass characterization and fifteen for the EPS extraction, to obtain three replicates from three mini-channels per block. Biofilms were scraped from these coupons with a sterile toothbrush and suspended in 15 mL (biomass) or in 100 mL (EPS) of culture medium before analysis. At the end of the disturbance sequence (day 32), and after the rewetting period (day 46), fifteen coupons were sampled in each of the three mini-channel and biofilm was suspended in 100 mL of culture medium for EPS extraction, to obtain three replicates per block.

### Biofilm Biomass

A subsample of the biofilm suspension obtained as described above was used to assess biofilm biomass by measuring the dry weight (DW) after 72 h of drying at 70°C in a pre-weighted aluminum dish.

### Maximum Quantum Yield

The physiological state of phototrophic microorganisms was estimated by the maximal quantum yield of the photosystem II (PSII) (yield, Φ PSII), corresponding to the number of open reaction centers (Rohacek and Bartak, 1999). In stressed organisms, the yield strongly decreases (Rohacek and Bartak, 1999). Though it is primarily linked to the PSII, the yield may reflect the physiological state of the organism (Parkhill et al., 2001; Laviale, 2008). The photosynthetic efficiency is specific to each algal group, precluding the comparison between the values of the different species used here. Non-invasive PhytoPAM measurements (Phytoplankton Analyzer, Heinz Walz GMBH, Effeltrich, Germany) were performed on the surface of the biofilm by measuring fluorescence response to a saturating light flash after 20 min of dark adaptation [dark-adapted state (DAS) conditions] (Baker, 2008):


(1) Φ(DASconditions)PSII=1-(F/0F)M;0<Φ<PSII1

F_M_ represents the maximal fluorescence and F_0_ corresponds to the minimal fluorescence of the photosystem II. For each condition (in one block), thirty-two measurements were performed at day 14 (eight for each mini-channel).

### Extracellular Polymeric Substances

#### Extracellular Polymeric Substances Extraction

The biofilm suspension was homogenized with an Ultra-Turrax disperser at 13,500 rpm for 1 min at room temperature. Then, a volume of the homogenized suspension corresponding to 10 mg DW was centrifuged at 4,000 *g* for 15 min. The pellet was washed with Phosphate Buffer Saline (PBS) solution and centrifuged again. Finally, two EPS extraction steps, following the sequential method previously developed by Loustau et al. (2018), were applied in sequence with intermediate centrifugations (4,000 *g* for 15 min) to collect the supernatants containing the solubilized EPS. The first extraction step was performed with 0.22% formamide in PBS solution (stirring at 150 rpm and 4°C for 60 min) and the second one with cation exchange resin (CER, Dowex Marathon) at 50 g g^–1^ DW of biomass in PBS solution (stirring at 150 rpm and 4°C for 90 min).

The cellular integrity during the extraction steps was controlled by the measurement of chlorophyll *a* released in the supernatants, giving a cell lysis level less than 3.5% for all samples.

The total amount of EPS extracted was the sum of the EPS amounts contained in both supernatants (formamide + CER). After acidification of EPS extract with hydrochloric acid and volatilization of inorganic carbon by catalytic oxidation at 720°C, the Total Organic Carbon (TOC) was analyzed (Shimatzu TOC-L analyzer).

#### Proteins and Polysaccharides Quantification

For each EPS extract, the amounts of proteins and polysaccharides were measured in analytical triplicates. Protein (PN) concentration was measured by the bicinchoninic acid (BCA) method (Smith et al., 1985). Twenty-five μL of EPS extract and 200 μL of BCA reagent (Sigma-Aldrich) were incubated in a 96-well microplate (15 min at 60°C) before measuring the optical density at 540 nm (microplate reader, Synergy Mx Biotek). Bovine serum albumin (BSA) with concentrations ranging from 0 to 800 mg L^–1^ was used as a standard. Polysaccharide (PS) concentration was measured by the anthrone method (Dreywood, 1946). One hundred μL of EPS extract and 200 μL of anthrone reagent (2% anthrone in 96% sulfuric acid) were incubated in a 96-well microplate (30 min at 60°C), cooled at room temperature for 10 min before measuring the optical density at 620 nm. Glucose with concentrations ranging from 0 to 100 mg L^–1^ was used as a standard.

### Statistical Analyses

The pre-disturbance state was used as a reference to assess the impact of the disturbance sequence and the recovery after rewetting on EPS production (quantity and quality). The measurements realized at day 32 (D32, end of disturbance) and day 46 (D46, end of re-wetting period) were normalized by the measures realized at day 14 (D14, before disturbance), by the following expressions:


(2) Impact(%)=100-(100×V/D32V)D14

(3) Recovery(%)=100×V/D46V14D

where V_D__14_ represents the mean value of the parameter for the measurements performed from one block (including three mini-channels sampled) at day 14, and V_D__32_ and V_D__46_ represent the mean value of the same parameter for the measures performed from one mini-channel of the same block at day 32 and day 46, respectively.

Statistical analyses were performed using Statistica software (version 8). For all analyses, the normality and homoscedasticity were checked on each dataset (biofilm biomass, photosynthetic efficiency, EPS amount) with the Shapiro-Wilk test and data were transformed if needed. Two-way ANOVAs and one-way ANOVAs were used to test the effects of environmental factors (temperature and light intensity or P concentration) (*i*) on biofilm biomass, photosynthetic efficiency and EPS amount and composition before the disturbance or (*ii*) on the impact and recovery of EPS amount and composition after the disturbance and the rewetting periods, respectively, followed by a Tukey *post hoc* test. The magnitude of the difference between pre- and post-disturbance (end of disturbance or end of rewetting period) was assessed using Hedges’g (Hedges, 1981) effect size calculated from *t*-statistics derived from student tests (Berben et al., 2012) using the R package {esc}. The magnitude of the effects of temperature, light and phosphorous concentration was measured using the partial eta-squared effect size calculated from 2-way and one-way ANOVAs with the R package {effectsize}. Data given in the text are means ± standard error (SE). For all statistical analyses, significance was inferred at *p* < 0.05.

## Results

### Combined Effects of Light Intensity, Temperature, and Phosphorus on Biofilm Physiology and Extracellular Polymeric Substances Production Before the Sequential Disturbance

The effects of light intensity, temperature, and P concentration were considered during the first 14-day period on biofilm (growth and maximum quantum yield) and EPS production (quantity as extracted TOC and quality as PN/PS ratio) (Table 1). The two biofilms showed very contrasted responses to the environmental conditions, with a higher influence of temperature on the diatom biofilm and a higher influence of light intensity and phosphorus concentration on the green alga biofilm.

**TABLE 1:** *P*-values for two-way and one-way ANOVAS to test the effect of environmental conditions on the biomass (DW), maximum quantum yield (ΦPSII), EPS quantity (TOC), and EPS quality (PN/PS) of the biofilms before the disturbance.

		**Temperature**	**Light intensity**	**Temp ^∗^ Light**	**P concentration**
*N. palea* biofilm	Biomass	**0.0256**	0.5431	0.6095	0.0854
	ΦPSII	**0.000001**	**0.0008**	**0.0461**	**0.0042**
	TOC	**0.0006**	0.8876	0.5189	**0.0004**
	PN/PS	0.0569	0.0777	0.0564	**0.0481**
*U. confervicolum* biofilm	Biomass	**0.0011**	**0.000002**	0.4683	**0.0010**
	ΦPSII	0.0549	0.1909	0.1909	**0.0496**
	TOC	0.1989	**0.0062**	0.0991	0.0509
	PN/PS	**0.0011**	**0.000002**	0.4683	**0.0047**

*Significant *p*-values are in bold, *n* = 3.*

**FIGURE 2:**
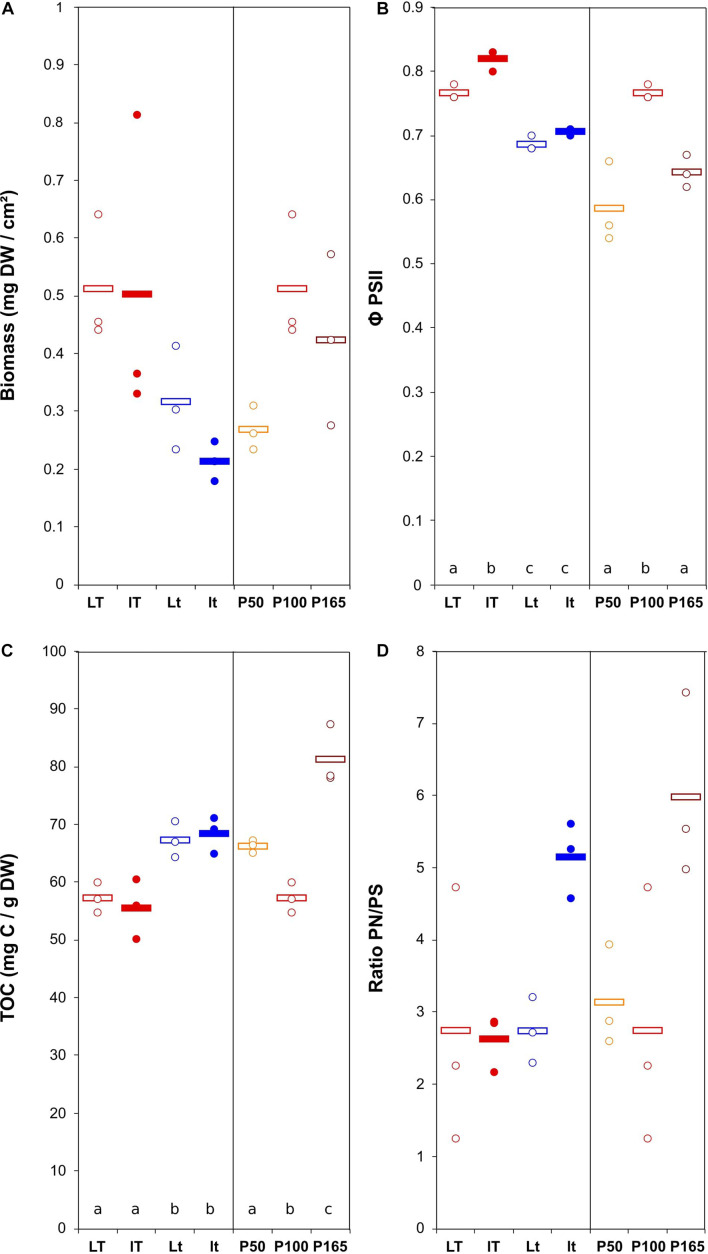
Scatter plot of the biomass (*DW* = dry weight) **(A)**, maximum quantum yield **(B)**, total organic carbon (quantity of EPS) **(C)**, and ratio protein/polysaccharide in the EPS **(D)** in *N. palea* biofilm before the disturbance. The letters indicate statistically homogeneous groups (Tukey *post hoc* test after ANOVA). No letters for non-significant ANOVA (the bars represent the means, *n* = 3). L/l: high/low light intensity (P100), *T*/*t*: high/low temperature (P100), P50, P100, P165: 50, 100, and 165% of the standard initial phosphorous concentration, respectively, associated with *L* and *T* conditions.

The biomass production varied from 0.21 ± 0.03 to 0.51 ± 0.06 mg DW cm^–2^ for *N. palea* biofilm, with a 2.4 ratio between the lowest and the highest mean values (Figure 2). The diatom growth and Φ PSII were positively affected by the temperature. Those parameters were less impacted by the light intensity. The modification of phosphorus concentration tented to decrease both growth and Φ PSII (Figure 2). The response of EPS production (TOC) to the environmental conditions was opposite to that of the biomass. The range of variation was narrower than for the biomass, with a 1.4 ratio between the lowest and the highest mean values. The ratio PN/PS was only significantly affected by P concentration, with the lowest value obtained at the lowest P concentration.

**FIGURE 3:**
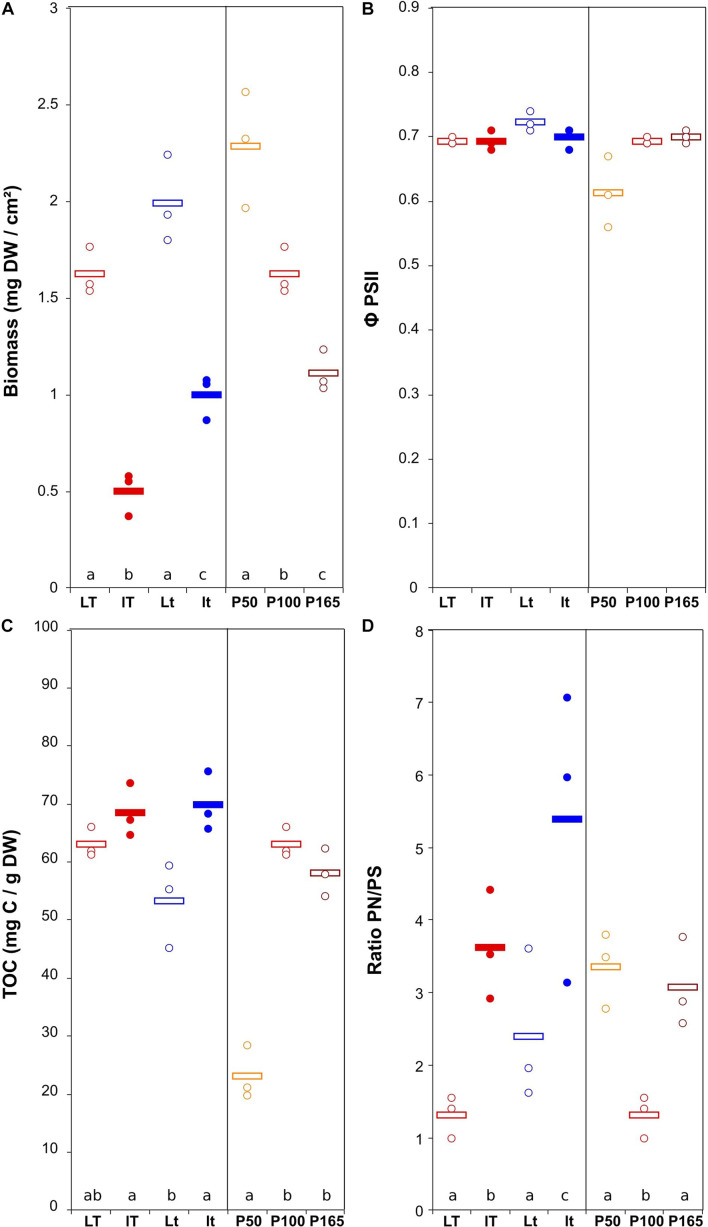
Scatter plot of the biomass (*DW* = dry weight) **(A)**, maximum quantum yield **(B)**, total organic carbon (quantity of EPS) **(C)**, and ratio protein/polysaccharide in the EPS **(D)** in *U. confervicolum* biofilm before the disturbance. The letters indicate statistically homogeneous groups (Tukey *post hoc* test after ANOVA). No letters for non-significant ANOVA (the bars represent the means, *n* = 3). *L*/*l*: high/low light intensity (P100), *T*/*t*: high/low temperature (P100), P50, P100, P165: 50, 100, and 165% of the standard initial phosphorous concentration, respectively, associated with *L* and *T* conditions.

The biomass production was more variable for the *U. confervicolum* biofilm, with mean values from 0.50 ± 0.06 to 2.29 ± 0.17 mg DW cm^–2^ (4.6 highest/lowest ratio) (Figure 3). It was positively influenced by light intensity and negatively affected by the temperature (Table 1). The biofilm growth was also negatively impacted by P concentration. The Φ PSII was only slightly modified by the environmental conditions. The same tendency was observed for the EPS production, with an additional negative effect of high light intensity. The PN/PS ratio in the green alga biofilm was very variable, with a 4.1 ratio between the highest and the lowest mean values. It was increased with a decrease of light intensity and temperature, and by a modification in P concentration compared to the reference condition.

To summarize these results, the increase in temperature yielded higher accumulation of diatom biofilm biomass and decreased EPS content and PN/PS ratio, while green alga biofilm accumulated more biomass at the highest light condition and exhibited lower EPS content and PN/PS ratio.

For the two biofilms, there was a significant negative correlation between biomass and EPS production (*r* = −0.49, *p* = 0.025 and *r* = −0.73, *p* < 0.001 for the diatom and green alga biofilms, respectively) (Supplementary Figure 1). For the diatom biofilm, EPS production was also negatively correlated with the maximum quantum yield (*r* = −0.70, *p* < 0.001). For the green alga biofilms, a positive correlation was observed (*r* = 0.63, *p* = 0.002), but it was driven by one treatment only. Without these points the correlation was similar to the one of the diatom biofilms (*r* = −0.47, *p* = 0.05).

### Impact of the Disturbance and Recovery of Extracellular Polymeric Substances Quantity and Quality

**FIGURE 4:**
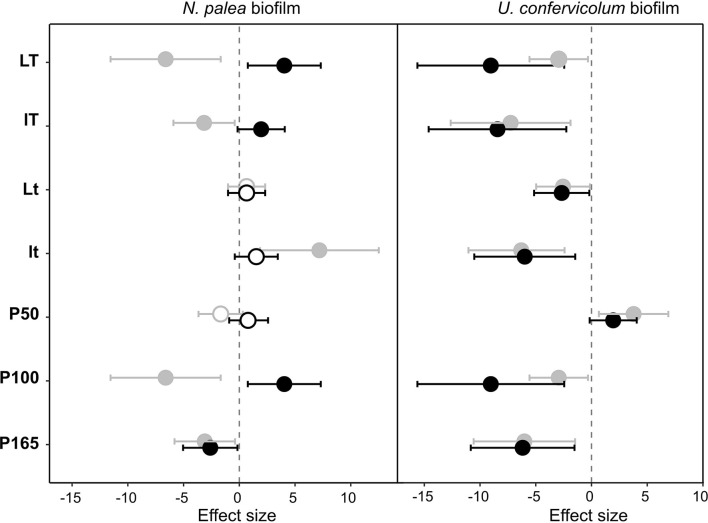
Effect size (Hedges’s ± 95% confidence interval) on the total organic carbon (EPS quantity) measured before the disturbance and at the end of the disturbance (gray circles) or before the disturbance and at the end of the rewetting period (black circles), for the *N. palea* biofilm and *U. confervicolum* biofilm. The effect size was measured for each growth condition. Full circles: significant effects, open circles: non-significant effects. *L*/*l*: high/low light intensity (P100), *T*/*t*: high/low temperature (P100), P50, P100, P165: 50, 100, and 165% of the standard initial phosphorous concentration, respectively, associated with *L* and *T* conditions.

**FIGURE 5:**
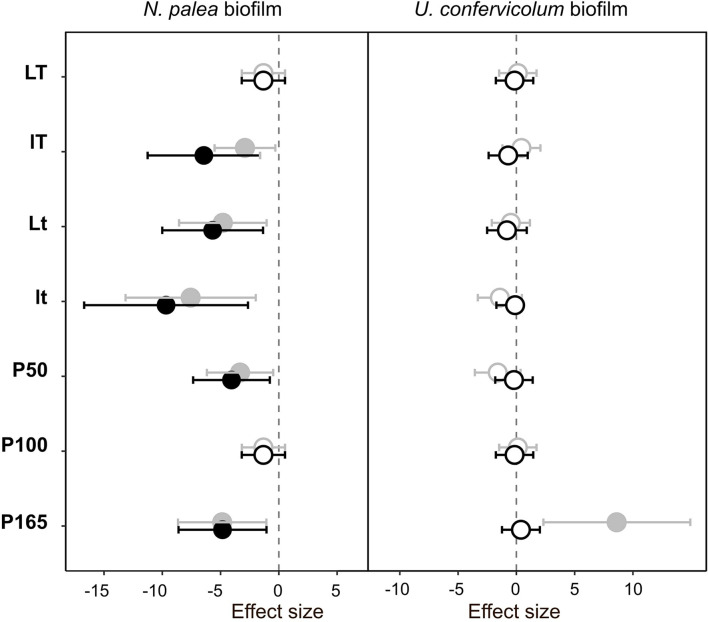
Effect size (Hedges’s ± 95% confidence interval) on the protein/polysaccharide ratio (EPS quality) measured before the disturbance and at the end of the disturbance (gray circles) or before the disturbance and at the end of the rewetting period (black circles), for the *N. palea* biofilm and *U. confervicolum* biofilm. The effect size was measured for each growth condition. Full circles: significant effects, open circles: non-significant effects. *L*/*l*: high/low light intensity (P100), *T*/*t*: high/low temperature (P100), P50, P100, P165: 50, 100, and 165% of the standard initial phosphorous concentration, respectively, associated with *L* and *T* conditions.

**FIGURE 6:**
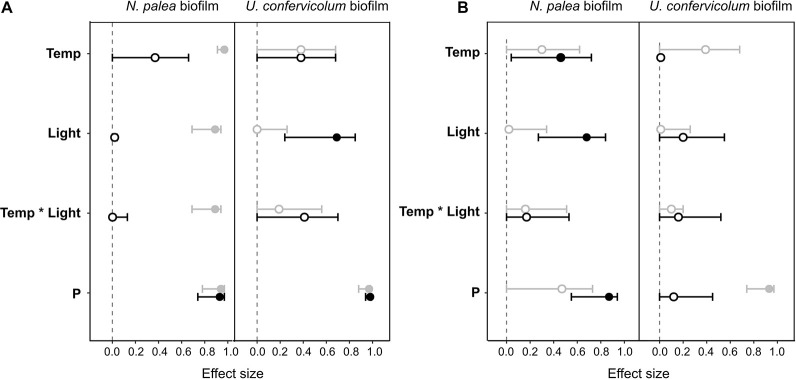
Effect size (partial eta-squared ± 95% confidence interval) on the impact (gray circles) or recovery (black circles) of the total organic carbon **(A)** and the protein/polysaccharide ratio **(B)** for the *N. palea* biofilm and *U. confervicolum* biofilm for the environmental factors tested: temperature (Temp), light intensity (Light), phosphorous concentration (P). The temperature ^∗^ light intensity interaction is also given. Closed circles: significant effects, open circles: non-significant effects.

The response of the biofilms to the disturbance, in terms of EPS quantity and quality, was analyzed in two steps. First, we compared pre- and post-disturbance values to show potential resistance and recovery for each environmental condition (Figures 4, 5). The post-disturbance values were either measured at the end of the disturbance (indication of resistance) or at the end of the rewetting period (indication of recovery). Second, we assessed the effects of light, temperature and P concentration on the resistance and resilience with ANOVA and derived effect size (Figure 6 and Table 2).

**TABLE 2:** *P*-values for two-way and one-way ANOVAS to test the effect of environmental conditions on the resistance and recovery of biofilm EPS quantity and quality.

			**Temperature**	**Light intensity**	**Light ^∗^ Temp**	**P concentration**
Resistance	*N. palea* biofilm	TOC	**0.000001**	**0.00004**	**0.00005**	**0.0002**
		PN/PS	0.1018	0.6656	0.2587	0.15000
	*U. confervicolum* biofilm	TOC	0.0950	0.7290	0.2400	**0.00004**
		PN/PS	0.0525	0.7690	0.3680	**0.0004**
Recovery	*N. palea* biofilm	TOC	0.0624	0.7152	0.9074	**0.0004**
		PN/PS	**0.0308**	**0.0033**	0.2344	**0.002**
	*U. confervicolum* biofilm	TOC	0.1114	**0.0043**	0.0637	**0.00001**
		PN/PS	0.7769	0.1984	0.2503	0.67

*Significant *p*-values are in bold, *n* = 3.*

The sequential disturbance had a strong impact on EPS quantity (Figure 4 and Supplementary Figures 1, 2). For the *N. palea* biofilm, it induced a decrease in EPS quantity, with the exception of the ltP100 condition (increase) and LtP100 and LTP50 conditions (no effect). For the *U. confervicolum* biofilm, the decrease in EPS quantity was observed in all conditions except the LTP50. For both biofilms, the increase in temperature increased the negative impact of the disturbance. For the *N. palea* biofilm, at the end of the rewetting period, the EPS quantity values were either identical or higher than the pre-disturbance values, while in the *U. confervicolum* biofilm they remained lower in all but one condition. For both algae, EPS quantity was less affected by the disturbance at low phosphorus concentration than at medium and high concentrations.

The quality of EPS was very sensitive to the combined disturbance in *N. palea* biofilm but only marginally affected in *U. confervicolum* biofilm (Figure 5 and Supplementary Figures 2, 3). The disturbance induced a decrease in the PN/PS ratio in the diatom biofilm in all conditions except the LTP100 one, where no effect was observed. Contrary to the EPS quantity, no recovery was observed at the end of the rewetting period for the EPS quality.

Combined effects of temperature, light, and phosphorous concentration on the resistance and recovery were assessed with ANOVAs (Table 2) and eta-squared effect sizes (Figure 6). For the impact of disturbance on EPS quantity (Figure 6A), all the environmental factors had a significant effect for the *N. palea* biofilm. When temperature, light intensity, and P concentration increased, the impact of the disturbance increased as well. In contrast, the environmental factors had no effect on the impact of disturbance on EPS quality (Figure 6B). For *U. confervicolum* biofilm, only the phosphorus concentration modified the impact of the disturbance on both EPS quantity and quality, with opposite directions: for the quantity the impact was lower at the lowest concentration, while for EPS quantity, the impact was lower at the highest concentration.

The recovery of EPS after the rewetting period was not influenced by the same factors than the resistance to the disturbance. For the *N. palea* biofilm, P concentration modified the recovery of both EPS quantity and quality, with the lowest resilience observed for the highest concentration. The recovery of EPS quality also increased with an increase in temperature and light intensity.

## Discussion

EPS are critical for the resilience of biofilms exposed to various stresses (Kroll et al., 2014; Flemming et al., 2016; Shetty et al., 2019). It is thus important that their production remains stable despite the disturbance biofilms may be exposed to. EPS production by biofilms is known to be influenced by environmental conditions (Wolfstein and Stal, 2002; Di Pippo et al., 2012). The question tackled here is in what extent variations in the environmental conditions could affect the resilience properties of EPS when biofilms are disturbed. A follow-up study would be to assess if modifications in biofilm EPS resilience can impact the functional resilience of biofilms. Our first hypothesis was confirmed, the results showing a strong influence of light, temperature, or phosphorous on the resilience of EPS quantity and quality. Contrary to our second hypothesis, the factors that influenced the most biofilm growth were not necessarily the factors influencing the EPS resilience. Moreover, factors that had no effects on biofilm growth or EPS production strongly influenced EPS resistance and recovery (e.g., light intensity for the *N. palea* biofilm).

Given that diatoms and green algae are well-known to produce high amount of EPS (de Brouwer et al., 2002; Xiao and Zheng, 2016; Bohorquez et al., 2017) and that the biomass of the algal cells accounted for the majority of biofilm biomass (microscopic observations), we assumed that the EPS found in the biofilms were mainly produced by algal cells. However, the analyses that were performed did not allow the distinction between algal and bacterial EPS.

### The Effects of Light Intensity, Temperature, and P Concentration on Biofilm Physiology and Extracellular Polymeric Substances Production

Despite the moderate amplitudes in the variations of the environmental conditions, strong impacts on biofilm and physiology have been observed. Although the study considered only two different simplified biofilms, it demonstrated very different patterns in the response to environmental conditions: the diatoms were more sensitive to the temperature, and the green algae were more sensitive to the light intensity. Such contrasting patterns are consistent with the conclusion of Falk et al. (1996) who stated that it is not possible to describe a general mechanism for photosynthetic adjustment to temperature that encompasses all phototrophic species because of genetic diversity and differential strategies in growth and development.

Unlike Di Pippo et al. (2012) conclusion, 30 μmol photons s^–1^m^–2^ in the present study provided sufficient light intensity for the development of the phototrophic biofilms. Light intensity, from 30 to 70 μmol photons s^–1^m^–2^, induced no significant effect for *N. palea* biofilm growth. For the green alga biofilm, and in agreement with Ylla et al. (2009), light intensity was the limiting parameter which control the growth. *U. confervicolum* is a filamentous species and forms a thick biofilm with streamers in the water at the biofilm surface that can create a self-shading effect and the light intensity can therefore quickly become a limiting factor during biofilm development (Rao, 2010; Villanueva et al., 2011). On the contrary, the pennate diatom forms a thin biofilm and the light can be accessed by most cells. These differences in algal morphology, associated with the higher biomass reached in *U. confervicolum* biofilms, may explain the differential response to light intensity increase.

Phosphorus which is often directly involved in eutrophication processes (Smith et al., 1999), had an effect on the two phototrophic biofilms with an increase in P concentration inducing a decrease of the green alga biofilm growth and an increase in the diatom biofilm growth. These results suggest that the composition of natural phototrophic biofilms should strongly depend on the P concentration in the surface water. The maximum quantum yield of the green alga biofilm was promoted by increasing P concentration in the culture medium, until it reached a plateau. In contrast, for the diatom biofilm, a decrease of the yield was observed for the highest P concentration, suggesting that a threshold was exceeded. The yield is known to be influenced by nutrient stress in algae (Parkhill et al., 2001). The P concentration used in the experiment was very high, but it was chosen according to the concentration in combo medium to keep similar growth conditions than the stock cultures. Note that the nutrient level was not adjusted during the experiments.

Concerning EPS production, weak negative correlations were observed between the maximum quantum yield and the quantity of TOC extracted. This result is rather unexpected, given that many authors have shown that EPS are released by cells concomitantly to oxygenic photosynthesis (Staats et al., 2000a) and primary production (Underwood and Paterson, 2003), assimilated to a metabolic response to stresses (Perkins et al., 2001; Orvain et al., 2003). However, there is no direct and clear link between the maximum quantum and the photosynthetic activity. The negative relationship between biofilm growth and EPS production suggests that when algal growth is limited and the photosynthesis is not, organic carbon produced through photosynthesis may be accumulated under the form of EPS, similarly to the Growth-Differentiation Balance Hypothesis for the synthesis of defense metabolites (Herms and Mattson, 1992). However, other regulation mechanisms are likely to be involved since EPS production was increased under low light intensity conditions for the green alga biofilm. Note that an increased EPS production in nutrient-limited cells has also been observed in marine diatoms (Staats et al., 2000b; Khandeparker and Bhosle, 2001).

In addition to EPS quantity, their nature was directly influenced by the environmental factors. In the two biofilms, the EPS were dominated by proteins (PN/PS ratio above 1) in all the growth conditions. EPS are often considered as essentially composed of polysaccharides, but a similar high content in proteins has been reported elsewhere, for instance for biofilms at the sediment surface, in freshwater (Gerbersdorf et al., 2009) and marine (Agogue et al., 2014) environments. The PN/PS ratio was highly variable in *U. confervicolum* biofilms. The PN/PS ratio significantly decreased (resulting from an increase of the quantity of polysaccharides) with an increase in temperature.

### The Effects of Light Intensity, Temperature, and P Concentration on Extracellular Polymeric Substances Response to the Disturbance

Although several studies considered the effects of environmental conditions on biofilm EPS quantity and quality, to our knowledge none assessed how these conditions could modulate the resistance and recovery of EPS after a disturbance. Here we showed, in agreement with our first hypothesis, that the resilience of EPS production after a complex disturbance, i.e., metal exposure followed by dry and rewetting periods, strongly depended on the environmental conditions. This dependency to the environmental conditions varied between the two simplified biofilms that were tested. Moreover, the environmental conditions impacted differently the quantitative and qualitative response of EPS to the disturbance. This opposition of the response pattern of EPS quantity and quality in the two biofilms indicates a decoupling of the regulation of those two parameters. Contrary to our second hypothesis, the environmental factors that affected the resilience of EPS production were not completely the same that affected biofilm growth, as assessed with our factorial design. Indeed, in diatom biofilms, light intensity had no effect on growth while it significantly modified the resistance of EPS quantity and the recovery of EPS quality (see Table 2). Algal species have developed various metabolic adaptations and defense systems to survive under variety of environmental stresses. A four-day metal exposure at non-toxic concentration followed a 2-week dry period was enough to cause changes in the EPS production by the two phototrophic biofilms. At the end of the disturbance, the quantity of EPS was either higher or lower than that before the disturbance, depending on growth conditions and on algal species.

The underlying idea of this experiment is that EPS resilience should contribute to biofilm functional resilience. Unfortunately, this could not directly be assessed because such experiments require the measurement of gross or net primary production. Concerning the protective role of EPS, contrasting results can be found in the literature, notably for the heavy metals. The latter can have either increasing (e.g., Miao et al., 2009) or nil effect (Mota et al., 2015) on EPS production while EPS supposedly contribute to the defense of the cells against metals. EPS are supposed to contribute to the protection of microorganisms against disturbance through different mechanisms. For instance, drying disturbance encompasses multiple stresses such as limitation of substrate and nutrient diffusion, which can be limited by the use of extracellular polymers as a C source by the cells. Inversely, EPS produced by filamentous cells form the viscous mucilage that surrounds cells (Underwood and Paterson, 2003) and probably play an important direct role in cell protection under unfavorable conditions. Microorganisms tolerate desiccation through a set of molecular mechanisms including the production of polysaccharides that form a reservoir for water and proteins thus capable to stabilize macromolecules and membranes. Extracellular polysaccharides, probably very hydrophilic, could be very efficient to retain interstitial pore water and protect phototrophic cells against desiccation. Only for the diatom biofilm, we observed modifications of EPS composition with an increase in polysaccharides at the end of the disturbance period. However, to verify the stability of this new status, it would have been necessary to follow rewetted biofilms during a longer period. This is in agreement with Orvain et al. (2003) who suggested that polysaccharides are involved in the formation of a protective wall, supplying hydrophilic and stabilizing properties that could convey important functions to the biofilm matrix in terms of resistance to environmental stresses.

The results of this study showed a divergence in the resilience ability of two very simplified phototrophic biofilms regarding to their EPS production. It appeared that in several cases small variations in the environmental conditions had no direct effect on EPS production, while exhibited a significant effect on the resistance and recovery of EPS production after a realistic disturbance. Though the experiment was performed with very simplified biofilms, under artificial conditions, it calls a need for further investigations which are necessary to anticipate the effect of a disturbance on microbial physiology in natural biofilms. The fact that the 3-degrees temperature increase amplified the impact of the disturbance on EPS quantity suggests the existence of non-additive effects between the contamination-dry period disturbance and warming. In a context of global change, this is a preoccupying observation, which highlights the importance of considering not only the direct effects of warming and climate change on the microbial communities, but also on their indirect effects on the ability of communities to cope with additional disturbances. During the last decade, the number of studies considering the resilience of biofilms increased, as well as the number of studies considering the responses of ecosystem components to combined stresses. However, the studies focusing on the modulation of ecosystem resilience ability by environmental conditions remain very scarce. In a context of global change, with an increasing frequency of disturbances, a considerable effort should be made in this direction.

## Data Availability Statement

The original contributions presented in the study are included in the article/Supplementary Material, further inquiries can be directed to the corresponding author.

## Author Contributions

EG-N, EL, JL, and J-LR designed the experiments. CB, EL, JF, J-LR, and QA contributed to the laboratory work and analyses. OO and OP realized Cu speciation calculation. EL and JL wrote the first manuscript, and then improved by the other authors.

## Conflict of Interest

The authors declare that the research was conducted in the absence of any commercial or financial relationships that could be construed as a potential conflict of interest.

## Publisher’s Note

All claims expressed in this article are solely those of the authors and do not necessarily represent those of their affiliated organizations, or those of the publisher, the editors and the reviewers. Any product that may be evaluated in this article, or claim that may be made by its manufacturer, is not guaranteed or endorsed by the publisher.
