# Supplementary material for: The Response of Extracellular Polymeric Substances Production by Phototrophic Biofilms to a Sequential Disturbance Strongly Depends on Environmental Conditions
Source: Front Microbiol. 2021 Oct 11;12:742027. doi: 10.3389/fmicb.2021.742027 (PMC8542934; doi:10.3389/fmicb.2021.742027)
Supplement: Supplementary file 1 [file Data_Sheet_1.docx]

**The response of EPS production by phototrophic biofilms to a sequential disturbance strongly depends on environmental conditions**

**Supplementary Material**

Figure 1:


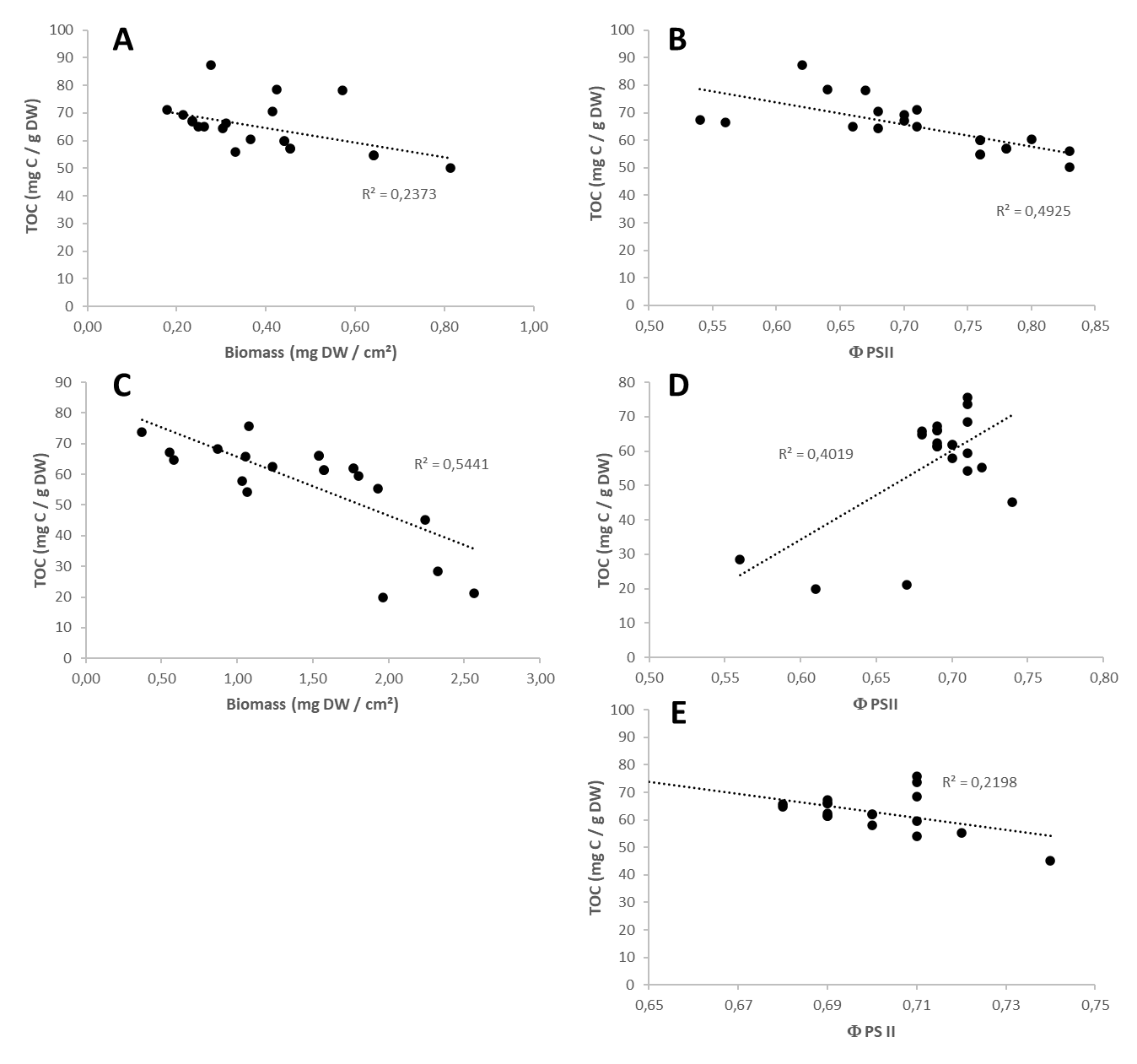


**Figure 1**. Correlations between the total organic carbon (EPS quantity) and the biomass **(A, C**) or the maximum quantum yield **(B, D, E)** before the disturbance, for the diatom biofilm **(A, B)** and the green alga biofilm **(C, D, E).** **(E)** is the same than **(D)**, but without the points corresponding to the condition LTP50. DW = dry weight.

Figure 2:


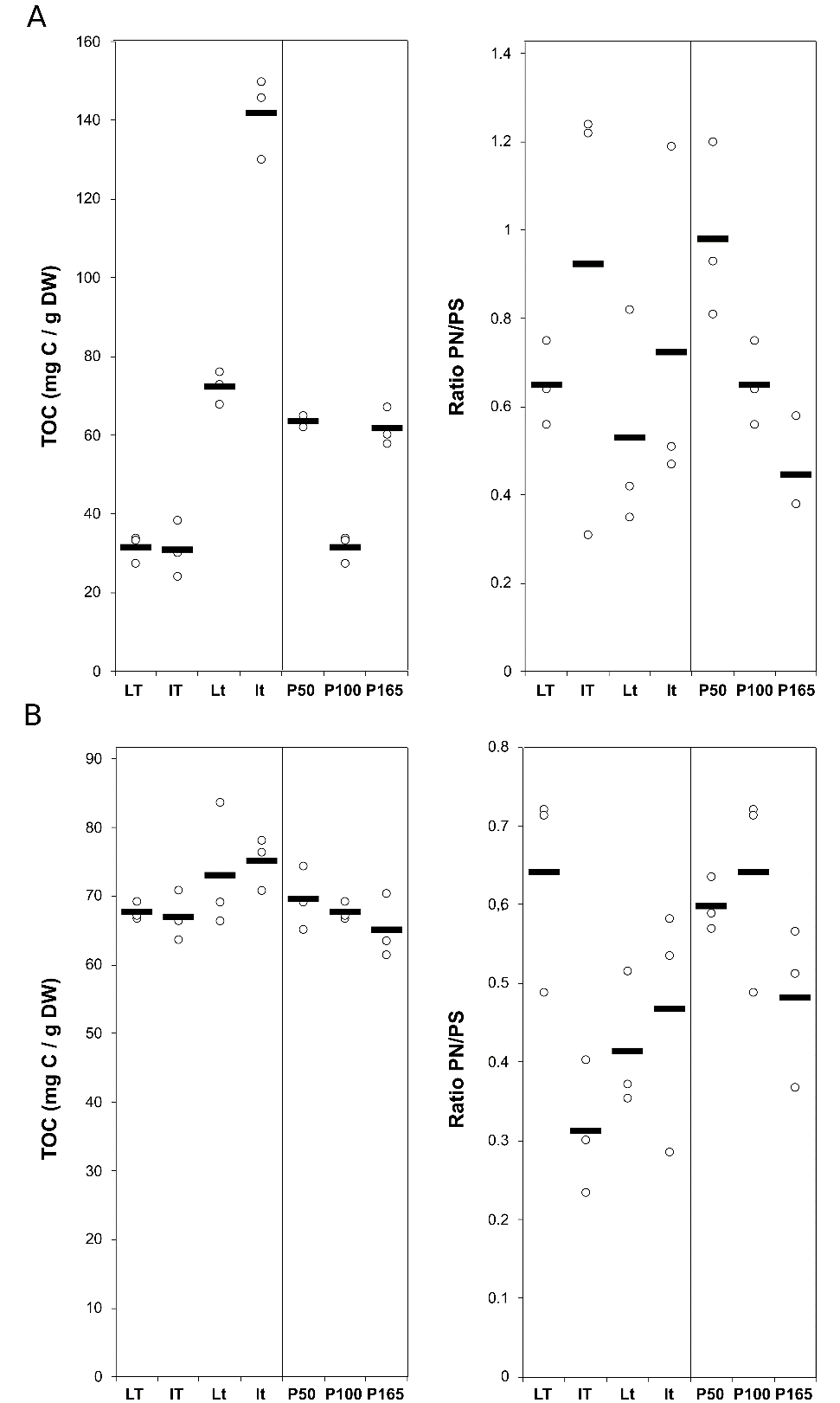


**Figure 2**. **(A)** Scatter plot of the total organic carbon (quantity of EPS) and ratio protein/polysaccharide in the EPS measured in *N. palea* biofilm at the end of the sequential disturbance. **(B)** Scatter plot of the total organic carbon and ratio protein/polysaccharide in the *N. palea* biofilm measured at the end of the rewetting period. The bars represent the means (n=3). L/l: high/low light intensity (P100), T/t: high/low temperature (P100), P50, P100, P165: 50%, 100% and 165% of the standard initial phosphorous concentration, respectively, associated with L and T conditions.

Figure 3:


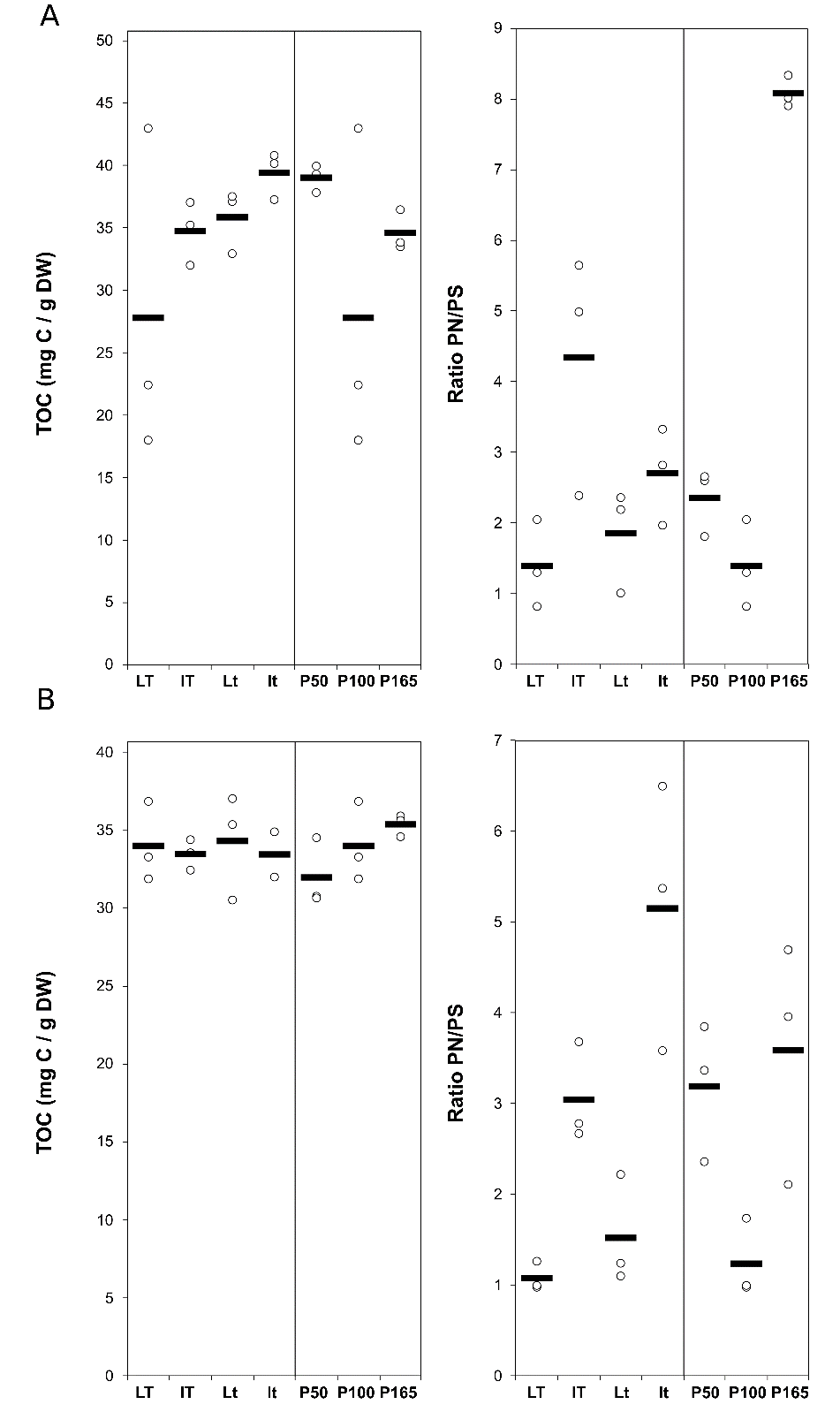


**Figure 3**. **(A)** Scatter plot of the total organic carbon (quantity of EPS) and ratio protein/polysaccharide in the EPS measured in *U. confervicolum* biofilm at the end of the sequential disturbance. **(B)** Scatter plot of the total organic carbon and ratio protein/polysaccharide in the *U. confervicolum* biofilm measured at the end of the rewetting period. The bars represent the means (n=3). L/l: high/low light intensity (P100), T/t: high/low temperature (P100), P50, P100, P165: 50%, 100% and 165% of the standard initial phosphorous concentration, respectively, associated with L and T conditions.
